# Genotype determination for polymorphisms in linkage disequilibrium

**DOI:** 10.1186/1471-2105-10-63

**Published:** 2009-02-20

**Authors:** Zhaoxia Yu, Chad Garner, Argyrios Ziogas, Hoda Anton-Culver, Daniel J Schaid

**Affiliations:** 1 Department of Statistics, University of California, Irvine, CA, USA; 2 Epidemiology Division, Department of Medicine, University of California, Irvine, CA, USA; 3 Division of Biostatistics, Department of Health Sciences Research, Mayo Clinic, Rochester, MN, USA

## Abstract

**Background:**

Genome-wide association studies with single nucleotide polymorphisms (SNPs) show great promise to identify genetic determinants of complex human traits. In current analyses, genotype calling and imputation of missing genotypes are usually considered as two separated tasks. The genotypes of SNPs are first determined one at a time from allele signal intensities. Then the missing genotypes, i.e., no-calls caused by not perfectly separated signal clouds, are imputed based on the linkage disequilibrium (LD) between multiple SNPs. Although many statistical methods have been developed to improve either genotype calling or imputation of missing genotypes, treating the two steps independently can lead to loss of genetic information.

**Results:**

We propose a novel genotype calling framework. In this framework, we consider the signal intensities and underlying LD structure of SNPs simultaneously by estimating both cluster parameters and haplotype frequencies. As a result, our new method outperforms some existing algorithms in terms of both call rates and genotyping accuracy. Our studies also suggest that jointly analyzing multiple SNPs in LD provides more accurate estimation of haplotypes than haplotype reconstruction methods that only use called genotypes.

**Conclusion:**

Our study demonstrates that jointly analyzing signal intensities and LD structure of multiple SNPs is a better way to determine genotypes and estimate LD parameters.

## Background

Recent advances in genotyping technologies have greatly improved genotype call rates and accuracy. This consequently enhances our understanding of genetic variations that are responsible for complex human traits. In particular, studies with SNPs show great promise to identify genetic determinants of complex disorders. Despite these achievements, the large number of SNPs in today's genome-wide studies poses a number of serious challenges. For example, one important issue is how to handle missing genotypes. Simply excluding subjects that have any missing genotypes is impractical in many situations, as most subjects usually have one or more missing genotypes in large genotyping efforts. Consequently, ignoring missing genotypes may lead to huge loss of genetic information.

Genotypes of SNPs are usually determined one at a time based on signal intensities of alleles. And depending on the availability of training data, the assignment of genotypes is often treated as a clustering or classification problem. When the allele signal intensities of a SNP are not well separated, currently available genotype calling algorithms make no calls (so called missing values) on data points that are not assigned to a genotype cluster with a high posterior probability. Many strategies have been exploited to reduce the impact of missing genotypes on association analyses. One of them is to take genotype uncertainties into account when conducting association tests. For example, Plagnol *et al*. [1] treated posterior probabilities as weights of genotype assignments and used a weighted score statistic, and Kang *et al*. [2] incorporated genotype uncertainties into their haplotype estimation algorithm. Another strategy is to impute missing genotypes from called genotypes. For example, Souverein *et al*. [3] modelled a SNP and markers that are in LD with the SNP using the polytomous logistic regression model, and Dai *et al*. [4] used a classification tree method. A detailed comparison of several of those imputation methods can be found in Yu and Schaid [5]. Imputation of missing genotypes can also be a by-product of haplotype reconstruction, which estimates missing genotypes and unknown haplotype phase simultaneously [6-18]. All those imputation strategies are based on the fact that when SNPs are in LD, the unobserved genotypes can be imputed accurately based on genotypes observed at other SNPs.

Essentially, the above described approaches try to use information from two different perspectives: incorporating genotype uncertainty into association tests focuses on making full use of information from the signal intensities of each SNP, and imputing missing genotypes based on LD focuses on borrowing information from neighbouring SNPs. As each of them uses only partial information from the data, we would expect that genotype determination can be greatly improved in an approach that takes advantage of both "fuzzy" call and imputation of missing values on called genotypes. Here, we propose a new method that uses both signal intensities and LD information, with the two parts connected by jointly estimating the underlying cluster parameters and haplotype frequencies for multiple markers.

**Figure 1:**
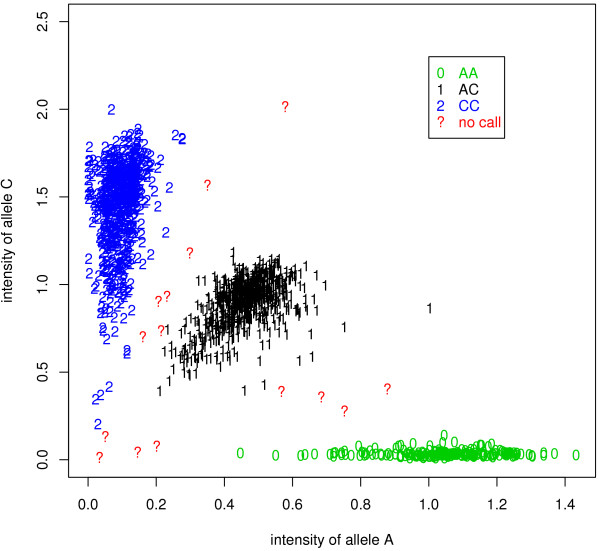
**The clustering results based on a one-marker-at-a-time method**. Values on the X-axis and Y-axis are normalized signal intensities of two alternative alleles (A and C). Estimated genotypes "AA", "AC", and "CC" are indicated by symbols "0", "1", and "2", respectively. Question marks represent missing values (i.e. no calls).

To show better the motivation for combining signal intensities and LD information, let's examine the clustering results of the signal data from Illumina BeadArray platform for about 1500 individuals at a SNP. Figure 1 shows the normalized signal intensities of two alternative alleles: A and C. Using the genotype calling algorithm *illuminus *[19], most data points can be clustered into one of three genotype groups: AA, AC, or CC. However, there are several data points that are located between two clusters. These data points are usually treated as missing values and their genotypes have to be imputed by some statistical methods. Despite the genotype uncertainty, the positions of these data points nevertheless provide some information for genotyping. For example, if the posterior probability for a data point to have genotype AA is 0.8, then it will be treated as a missing value when the threshold to assign genotype calls is set to 0.95; however, in the case that it is in LD with a neighbouring SNP, this partial genotype information, together with the LD between the two SNPs, can help to identify its underlying genotype.

## Methods

### Notation

***N***: number of subjects.

***M***: number of markers.

***H***: number of distinguishable haplotypes.

*X*_*i *_= (*X*_*i1*_, ..., *X*_*iM*_): vector of signal intensities for subject *i*, *i *= 1, ..., *N*.

*G*_*i *_= (*G*_*i1*_, ..., *G*_*iM*_): genotype for subject *i*, *i *= 1, ..., *N*.

*h*_*j *_= (*h*_*j1*_, ..., *h*_*jM*_): the *j *th haplotype, *j *= *1,...,H*. *h*_*jm *_is 1 for the rare allele and 0 for the common allele at SNP *m*.

*Y*_*i *_= *(h*^*i1*^/*h*^*i2*^): phased genotype (haplotype pairs) for subject *i*, where *h*^*i1 *^∈ {*h*_*1*_,...,*h*_*H*_} and *h*^*i2 *^∈ {*h*_*1*_,...,*h*_*H*_} are the haplotypes. *i *= 1, ..., *N*.

*h*_*j*_(*Y*_*i*_): number of copies of haplotype *h*_*j *_in *Y*_*i*_.

*θ*_*S *_= (*μ*_*mt*_, *Σ*_*mt*_, *π*_*mt*_)_*m *= 1,...,*M *_: cluster parameters, where *μ*_*mt*_, *Σ*_*mt*_, *π*_*mt *_are the mean, covariance matrix (or variance for one-dimensional data), and probability of genotype cluster *t *at SNP *m*, respectively.

*θ*_*H *_= (*θ*_*h1*_,...,*θ*_*hH*_): LD parameters, where *θ*_*hj *_is the frequency of haplotype *h*_*j*_.

In the notation, *X*_*i *_is observed, and *G*_*i *_and *Y*_*i *_are unobserved. *X*_*i *_= (*X*_*i1*_, ..., *X*_*iM*_) denotes signal intensities that are after proper normalization or transformation. Different normalization or transformation methods may be used for data generated by different platforms. For example, for signal data from Illumina BeadArray, *X*_*im *_is the contrast variable at SNP *m*. Similar to the algorithm *illuminus *by Teo *et al*[19], we define contrast as the ratio of the difference to the sum of the normalized signal intensities of two alternative alleles. For data from Affymetrix GeneChip 500 K arrays, *X*_*im *_is a two dimensional variable, with each dimension corresponding to the normalized signal of one allele, as in the *Chiamo *[20] software.

### Our new method

In our method we partition parameters into two sets: the cluster parameters *θ*_*S*_, and the LD parameters *θ*_*H*_. Although they contain redundant information, i.e., all *π*_*mt *_are known when *θ*_*H *_is given, we adopt this parameterization to simplify the presentation of our algorithm. Our algorithm correlates the signal intensities of multiple markers by jointly estimating cluster parameters and LD parameters (i.e., haplotype frequencies). We assume signal intensities of different markers are independent, conditional on given genotypes. In addition, we make three reasonable assumptions:

(1) Conditional on phased genotypes and cluster parameters, the distribution of signal intensities does not rely on LD parameters *θ*_*H *_i.e., *f*(*X*_*i*_|*Y*_*i*_, *θ*_*S*_, *θ*_*H*_) = *f*(*X*_*i*_|*Y*_*i*_, *θ*_*S*_);

(2) Conditional on LD parameters *θ*_*H *_the distribution of *Y*_*i *_does not depend on cluster parameters *θ*_*S*_, i.e., *f*(*Y*_*i*_|*θ*_*S*_, *θ*_*H*_) = *f*(*Y*_*i*_|*θ*_*H*_);

(3) SNPs are in Hardy-Weinberg equilibrium (HWE).

The complete data likelihood is then:

*f*(*X*_*i*_, *Y*_*i *_| *θ*_*S*_, *θ*_*H*_) = *f*(*X*_*i *_| *Y*_*i*_, *θ*_*S*_, *θ*_*H*_)*f*(*Y*_*i *_| *θ*_*S*_, *θ*_*H*_) = *f*(*X*_*i *_| *Y*_*i*_, *θ*_*S*_)*f*(*Y*_*i *_| *θ*_*H*_)

We use the EM algorithm to estimate parameters in our method [21]. With some algebraic manipulation, we can show that the E step is equivalent to calculating the genotype posterior probability for the *i*th subject to have genotype *t *at the *m*th locus, given estimated parameters and observed signal intensities. Based on the relation between phased and unphased genotypes, the genotype posterior probability can be expressed as

$$w_{imt}=\mathrm{Pr}(G_{im}=t|X_{i},\theta_{S},\theta_{H})=\sum_{Y_{i}:G_{im}=t}f(Y_{i}|X_{i},\theta_{S},\theta_{H})$$

This is essentially a sum of probabilities of phased genotypes, conditional on estimated parameters and observed signal intensities. The probability of a phased genotype conditional on estimated parameters and observed signal intensities is

$$f(Y_{i}|X_{i},\theta_{S},\theta_{H})=\frac{f(X_{i}|Y_{i},\theta_{S})f(Y_{i}|\theta_{H})}{\sum_{Y_{i}}f(X_{i}|Y_{i},\theta_{S})f(Y_{i}|\theta_{H})}$$

where

$$\begin{array}{l} f(X_{i}|Y_{i},\theta_{S})=\prod_{m=1}^{M}f(X_{im}|Y_{i},\theta_{S})\\ f(Y_{i}|\theta_{H})=\theta_{h^{i1}}\theta_{h^{i2}}(1+I(h^{i1}=h^{i2})) \end{array}$$

For given *Y*_*i *_and *θ*_*S*_, we assume that the signal intensities at different markers are independent, and they follow a distribution suitable to the normalized signal data, such as Gaussian distribution or *t*-distribution.

The M step in the EM algorithm calculates the set of *θ*_*S *_and *θ*_*H *_that maximizes the expected log-likelihood. The estimate for the frequency of haplotype *h*_*j *_is

$$\theta_{h_{j}}=\frac{1}{2n}\sum_{i=1}^{n}\sum_{Y_{i}}f(Y_{i}|X_{i},\theta_{S},\theta_{H})h_{j}(Y_{i})$$

Since *θ*_*S *_and *θ*_*H *_contain redundant information, there is no need to update the *π*_*mt*_'s in *θ*_*S *_once *θ*_*H *_is updated; as a result, we only need to estimate the mean and covariance matrix of each genotype cluster. In the M step of the conventional EM algorithm, the means and covariance matrices are updated by the set of values that maximizes the expected log-likelihood. For Illumina BeadArray data, our estimators for cluster parameters are based on data points with large genotype posterior probabilities (for example, greater than 0.95), since our study shows they are more robust than the estimators in the M step of the conventional EM algorithm. This strategy is also used in [19]. For data generated by the Affymetrix GeneChip 500 K arrays, we first obtained the regular estimates for the mean and covariance matrix of the *t*th cluster at SNP *m*, then estimated the cluster mean *μ*_*mt *_by using the (*N *× *π*_*mt*_/2) data points that have the smallest Mahalanobis distances, and finally updated the covariance matrix *Σ*_*mt *_by a weighted estimator based on estimated *μ*_*mt *_and all data points.

In our method, once the EM algorithm converges, we make genotype calls based on the genotype posterior probabilities and a predetermined threshold *δ*. Specifically, for a subject at a given SNP, if the posterior probability for the subject to have genotype *t *is greater than *δ*, we assign *t *to the corresponding data point; if the posterior probabilities for all of the three genotypes are less than *δ*, then the data point is labelled as missing and no call is assigned.

We emphasize that our method is different from treating signal intensities of different markers as a multi-dimensional variable. To show the difference, here we consider the case of two SNPs. The number of possible genotypes is nine for two SNPs. If we treat the contrast variable of the two SNPs as a bivariate *t*-distribution with known degrees of freedom, then we need to estimate 53 parameters, including nine two-dimensional means, nine two-by-two covariance matrices (three parameters in each covariance matrix), and eight cluster probabilities. By contrast, we only need to estimate 15 parameters in our method: six means, attributed to three genotype clusters for each SNP; six variances, attributed to three genotype clusters for each SNP; and three haplotype frequencies, attributed to four possible haplotypes. In addition, the cluster parameters in our method are estimated marker by marker; as a result, the difficulty brought by a SNP with a rare allele or low signal quality has little effect on the cluster parameters of other SNPs.

To assess the performance of our method (denoted as M3), we compared it with two other methods: the one-SNP-at-a-time method M1 and the LD based method M2. The method M1 models the cluster parameters *θ*_*S*_, and the method M2 models the LD parameters *θ*_*H*_. M1 and M2 use only part of the information from data: either the signal intensities of individual SNPs, or LD structure of multiple SNPs. By contrast, our method M3 uses both signal intensities of individual SNPs and LD structure between SNPs. In the method M1, we used the algorithm *illuminus *[19] for data from Illumina BeadArray and the algorithm *Chiamo *[20] for data from Affymetrix GeneChip 500 K arrays. Those two algorithms are used because they outperform other existing algorithms for their respective platforms [19,20]. The *illuminus *algorithm assumes that the contrast and strength (the natural logarithm of the sum of normalized signal intensities) follow a *t*-mixture model and it uses the EM algorithm  to estimate cluster parameters; the *Chiamo *algorithm uses a Bayesian hierarchical mixture model to call genotypes from normalized signal intensities. In the method M2, we first computed the posterior genotype probabilities for the missing genotypes based on the LD structure of genotypes assigned by *illuminus *or *Chiamo*, then assigned genotype calls to the missing ones based on whether the calculated posterior probabilities were greater than a threshold. The posterior genotype probabilities are calculated by using the EM algorithm [6] for our simulated data with two SNPs, and they are calculated by using the algorithm *fastPHASE *[11] for the two real data sets considered in the paper, due to the large number of SNPs in the real data sets.

### Simulated data

To evaluate the performance of different methods, we simulated data by mimicking signals generated by Illumina BeadArray. In our simulation, the cluster parameters were based on SNPs in a real data set; the allele frequencies and LD levels were pre-determined to reflect different data structures. We considered two SNPs with the same allele frequencies. The minor allele frequency was chosen to be 0.05, 0.1, or 0.3, and the LD was chosen at a variety of levels, with the square of the Pearson's correlation (*r*^2^) being about 0, 0.3, 0.5, or 0.8. HWE was assumed for both SNPs. The threshold for assigning calls or no calls, *δ*, was chosen to be 0.85, 0.90, 0.95, or 0.975. The signal intensities (contrast and strength) for a given marker were assumed to have a three-component *t*-mixture distribution (bivariate), with six degrees of freedom. Contrast and strength were assumed to be independent from each other. To study the effect of signal quality, we considered three sets of cluster parameters:

(1) high quality, with means for contrast being -0.9, 0.2, 0.9, means for strength being -1.2, -1, -0.8, standard errors for contrast being 0.09, 0.09, 0.06, and standard errors for strength being 0.37, 0.22, 0.27;

(2) low quality, with means for contrast being -0.5, 0.3, 0.9, means for strength being -1.4, -1.2, -0.8, standard errors for contrast being 0.22, 0.15, 0.06, and standard errors for strength being 0.56, 0.64, 0.48;

(3) mixed quality, a situation with one SNP in high quality and the other one in low quality.

These parameters were taken from two SNPs in real data, representing SNPs with high or low genotype call rates. The sample size was 1500.

To quantify the performance of the methods, two quantitative metrics are computed: call rate and genotyping error rate. The call rate was the number of non-missing calls divided by the total number of calls, and the genotyping error rate was the proportion of incorrectly called genotypes among all assigned calls. For a set of chosen parameters, we used 1000 replicates to estimate the two metrics. As both the LD based method M2 and our method M3 estimate haplotype frequencies, it is interesting to investigate whether one method gives more accurate estimation for haplotype frequencies. And for two SNPs, the accuracy of haplotype frequencies of the two methods can be reflected by the square of the Pearson's correlation (*r*^2^) based on estimated haplotype frequencies.

### Real data

We considered two sets of real data from two different genotyping platforms.

The first data set consists of 1599 unrelated case-control subjects (one third cases and two thirds controls) from a breast-cancer candidate-gene study conducted in the University of California at Irvine. For each subject, 1455 SNPs in 148 candidate genes were genotyped by Illumina BeadArray. Normalized signal data were obtained from BeadStudio. We used the algorithm *illuminus *to make genotype calls in the one-SNP-at-a-time method M1. In the LD based method M2, due to the large number of SNPs, we used the algorithm *fastPHASE *to compute the posterior probabilities for the missing genotypes (no calls) assigned by the algorithm *illuminus*. Jointly analyzing the signal data of all the 1455 SNPs is computationally prohibitive. To solve that problem, for each SNP, we searched all other SNPs to find the one with the maximum *r*^2 ^with the given SNP, then analyzed the two SNPs jointly if their *r*^2 ^is greater than 0.3. A *t*-mixture model with fixed degrees of freedom was then used to cluster the contrast of two alternative alleles for each SNP. In all the three methods (M1, M2, and M3), we used 0.95 as the threshold to assign genotype calls.

The second data set consists of signal data from Affymetrix GeneChip 500 K arrays for 1504 unrelated controls from the 1958 British Birth Cohort [20], obtained from the Wellcome Trust Case Control Consortium (WTCCC). We focused our study on the 6277 SNPs in chromosome 22. We chose the algorithm *Chiamo *as the one-SNP-at-a-time method M1, since it outperforms other genotype calling methods [20]. This method uses a Bayesian hierarchical mixture model to cluster quantile normalized data, and its default threshold of posterior probabilities for making a call is 0.9. For the no-calls assigned by *Chiamo*, we imputed them using the algorithm *fastPHASE*. The joint analysis of signal and LD was similar to that applied to the first real data set, except that here we used a Gaussian mixture model for the two-dimensional signal data. To compare the performance of different methods, we downloaded the Illumina Infinium 15 K data from the WTCCC. Among the 1504 subjects with Affymetirx 500 K data, 1457 of them were also genotyped by the Illumina Infinium 15 K. And the number of common SNPs on chromosome 22 in the two platforms was 31. For direct comparison, in each method, we computed the discordance rates between the most probable calls at the 31 common SNPs. The reason why we use the most probable call for each data point is that the thresholds for different methods and platforms are not comparable.

## Results

### Simulated data

The results for the simulated data are reported in Tables 1 to 4. They are mean values from 1000 replications. Tables 1, 2, and 3 show the call rates and genotyping error rates of the three methods when (1) both of the two SNPs were in high quality, (2) both of the two SNPs were in low quality, and (3) SNP_1 _was in high quality while SNP_2 _was in low quality, respectively. Table 4 reports the estimated *r*^2 ^and mean square errors. Since the patterns of the results for different allele frequencies *p *were similar, here we only show the results for *p *= 0.1. Our study indicates that different thresholds *δ *led to very similar conclusions, thus only those of *δ *= 0.95 are described. And the results for *r*^2 ^= 0 are not shown, because when *r*^2 ^was close to 0, the performance of all the three methods were very similar, as expected.

**Table 1:** Call rate and genotyping error for two SNPs in high quality

	call rate (%)			error rate (%)		
*r*^2^	0.802	0.500	0.304	0.802	0.500	0.304

M1	99.75	99.75	99.75	0.05	0.05	0.05

M2	99.87	99.75	99.75	0.06	0.05	0.05

M3	99.87	99.82	99.80	0.04	0.05	0.05

M1: the one-SNP-at-a-time method; M2: imputation based on estimated genotypes from M1 and LD structure; M3: our method that jointly analyzes the signal of two SNPs.

**Table 2:** Call rate and genotyping error for two SNPs in low quality

	call rate (%)			error rate (%)		
*r*^2^	0.802	0.500	0.304	0.802	0.500	0.304

M1	97.96	97.97	97.95	0.53	0.54	0.54

M2	98.90	97.97	97.95	0.64	0.54	0.54

M3	98.75	98.35	98.16	0.32	0.44	0.50

**Table 3:** Call rate and genotyping error for two SNPs in mixed quality

		call rate (%)			error rate (%)		
*r*^2^		0.802	0.500	0.304	0.802	0.500	0.304

M1	SNP_1_	99.75	99.75	99.74	0.05	0.05	0.05
	SNP_2_	97.96	97.98	97.97	0.54	0.54	0.53

M2	SNP_1_	99.87	99.75	99.74	0.06	0.05	0.05
	SNP_2_	98.89	98.02	97.97	0.63	0.56	0.53

M3	SNP_1_	99.87	99.82	99.79	0.04	0.05	0.05
	SNP_2_	98.81	98.37	98.19	0.30	0.43	0.49

SNP_1 _was in high quality and SNP_2 _was in low quality.

**Table 4:** Comparison of M2 and our method M3 on the estimation of *r*^2^

	*r*^2^	0.802	0.500	0.304
	
high quality	M2	.798 (4e-5)	.497 (3e-5)	.302 (2e-5)
	M3	.799 (3e-5)	.498 (2e-5)	.303 (1e-5)
low quality	M2	.757 (.002)	.469 (.001)	.283 (6e-4)
	M3	.770 (.001)	.478 (6e-4)	.291 (3e-4)

mixed quality	M2	.775 (8e-4)	.482 (4e-4)	.293 (2e-4)
	M3	.784 (4e-4)	.488 (2e-4)	.297 (1e-4)

The first row shows true values of the square of Pearson's correlation. Numbers in parentheses are mean square errors.

The call rates of the two LD based methods (M2, and M3) depend significantly on the LD level between the two SNPs. As the method M1 (*illuminus*) calls one SNP at a time, the LD level does not affect its call rates, which can be seen from the almost identical call rates at all LD levels. When the two SNPs were independent from each other, as expected, the call rates of the three methods were very similar (data not shown). When the two SNPs were in LD, our method M3 consistently improved the call rates of M1, and with the increase of LD, its advantage became more considerable. When LD was not very strong, such as when *r*^2 ^was below 0.5, the increment brought by M2 over M1 was very small; in contrast, our method still improved the call rates in the same situation.

Now we examine the effect of signal quality on the call rates. When the signal quality of both SNPs was high, all the three methods gave very high call rates and our method gave the highest, as illustrated in Table 1. When the signal quality of both SNPs was low, although the call rates of all methods were lower, as shown in Table 2, the benefit of using our method was more noticeable. To study if our method would improve the genotype calling of one SNP but deteriorate that of the other, we tested it on the simulated data of mixed quality, and the results are shown in Table 3. Here, because the two SNPs were assumed to have the same rare allele frequency, the signal intensities of the SNP in low quality were more difficult to cluster than those of the SNP in high quality. As a result, the call rates of M1 for SNP_2 _were much lower than those of SNP_1 _for all three methods. However, by jointly modelling signal intensities and LD, our method significantly increased the call rates of SNP_2_. In addition, the call rates of SNP_1 _were also increased. This might seem implausible at the first sight, but it can be explained by the fact that, although the signal intensities of SNP_2 _were noisy, they were nevertheless useful to help cluster the signal intensities of the associated marker SNP_1_. Consequently, jointly calling the two SNPs increased the call rates for both of them.

Now let's examine the other important metric – the genotyping error rate. For a given method with different thresholds, genotyping accuracy and call rates are often trade-off of each other. It is therefore important for an algorithm not to gain higher value of one at the cost of the other. The results in Tables 1, 2, and 3 illustrate that our method not only achieved greater call rates than M1, but also achieved higher genotyping accuracy, which is most obvious when LD was moderate or strong. By contrast, although the method M2 increased the call rates when LD was strong, this increase was gained at the sacrifice of genotyping accuracy. For example, when both SNPs were in low quality and *r*^2 ^was about 0.8, both M2 and our method have higher call rates than M1; however, the genotyping error rate of M2 was 0.64%, which was 2 times of the error rate of our method (0.32%) and 1.2 times of that of M1 (0.53%).

When the signal intensities of a SNP are in low quality, the estimated genotypes are prone to genotyping errors. This error was amplified by the method M2 (Tables 2 and 3), because M2 is directly based on called genotypes. By contrast, our method achieved higher genotyping accuracy. This is because, for a SNP whose signal intensities are not well separated, its genotype assignments can be improved by the LD between itself and other SNPs. Strikingly, in the situation of mixed quality, when the two SNPs were in high LD, our method improved the genotyping accuracy for both SNPs over the method M1. Again, this can be attributed to the fact that although the signal of SNP_2 _was noisy, it still provided some clustering information for SNP_1_.

Table 4 shows the estimated *r*^2 ^and mean square errors. With all LD levels and signal qualities, our method outperformed the method M2 by providing estimated correlations that were much closer to their true values, with smaller mean square errors. This is because our method gave better call rates and genotyping accuracy, and as a result, it provided more accurate estimation of *r*^2 ^and haplotype frequencies.

### Real data

For the data set of the breast-cancer candidate-gene study, we computed the call rates of the 1455 SNPs using all the three methods. The median call rates of M1 (*illuminus*), M2 (*fastPHASE*), and our method M3 were 98.75%, 98.87%, and 99.44%, respectively. Compared to the one-marker-at-a-time method *illuminus*, our method increased the call rates of 294 SNPs for at least 1%, and 13 SNPs for at least 5%. By contrast, *fastPHASE *increased the call rates of only 57 SNPs for at least 1%, and only 4 SNPs for at least 5%. Although there is no additional data to compare the genotype accuracy, the results here show convincingly that our method significantly improves the call rates of the other two methods.

In the WTCCC data, 6277 SNPs on chromosome 22 were genotyped for 1504 unrelated controls using the Affymetrix 500 K platform. Here we computed the discordance rates between the calls from the three methods and the calls from the Infinium 15 K. The discordance rates were 0.85% for *Chiamo*, 0.41% for *fastPHASE*, and 0.27% for our method. Those discordance rates were calculated based on the most probable calls. Among the data points with *Chiamo *scores below 0.9, i.e., the data points with "no calls" by *Chiamo *with its default threshold 0.9, the discordance rates of *fastPHASE *and our method were 27.74% and 14.23%, respectively. Note that the discordance rates for data points with low *Chiamo *scores were much higher than the overall discordance rates. We examined the pattern of *Chiamo *scores, and found that most of the data points with low scores were in a few SNPs that have relatively low call rates, which is likely an explanation for the high discordance rates. All those discordance rates indicate that our method increases the genotyping accuracy.

## Discussion and Conclusion

In existing analyses, genotype calling and imputation of missing genotypes are usually considered as two separate problems. In this paper, using simulated data and real data, we have demonstrated that it is advantageous to treat the two problems simultaneously. Currently, approaches based on LD between SNPs have been widely used to impute missing genotypes. Those approaches, however, might decrease genotyping accuracy, when comparing to the traditional one-marker-at-a-time genotype calling method. By contrast, our method outperforms the traditional one-marker-at-a-time method in the respects of both call rate and genotyping accuracy. Besides genotype calls, our method also yields a weight matrix consisting of genotype probabilities. This weight matrix can be used in association analyses, in a similar way described by Plagnol *et al *[1].

Although in our method SNPs are assumed to be in Hardy-Weinberg equilibrium, we found our method still improves the results of call rate and genotyping accuracy for data with moderate departure from HWE (data not shown). Another assumption we made is that subjects are from a random sample of a population, which means subjects are unrelated and there is no population stratification. In the future, we plan to modify our method so that related subjects with known pedigree structure and population substructure can also be taken into account.

Our method does not put any constraint on the number of SNPs that can be called jointly. However, because the number of haplotypes increases exponentially with the number of jointly-analyzed SNPs, it is computationally demanding for the traditional EM algorithm to process a large number of SNPs. To solve this problem, what we have done in the application of our method to the real data is that, for each SNP, we made genotype calls by using another SNP that had the highest Pearson's correlation with the original SNP. Although this significantly mitigates the computation requirement, it might not be the best strategy. An alternative way is to use a sliding window method to fix the number of SNPs that are called together; however, here we decide not to do that, because LD between SNPs is usually a localized phenomenon and the number of SNPs that are analyzed together should be determined by local LD structure. Other alternatives include several recently proposed methods that can provide rapid and accurate estimation of haplotypes [11,13-15].

What we considered here are signal intensities obtained from two different platforms: the Illumina BeadArray and the Affymetrix GeneChip 500 K arrays. There is no reason, however, to limit the method to those platforms. In fact, the idea of jointly analyzing signal intensities of multiple markers is quite general. It can be applied to other platforms, such as multiple inversion probe technology [22], and other DNA variations, such as copy number variants. The advantage of combining signal intensities and LD is confirmed by a few recent publications [23,24], where the signal data and dependence between neighbouring markers are integrated to infer copy numbers and loss of heterozygosity.

## Authors' contributions

ZY designed the study, developed the statistical method and computational tool; DJS was involved in designing the study; CG, ZA, and HA-C contributed one real data set. All authors were involved in writing the manuscript.
